# Success: the synergy of off-pump coronary artery bypass and living donor liver transplantation—a two-case report

**DOI:** 10.3389/fsurg.2025.1587370

**Published:** 2025-06-19

**Authors:** Sinan Efe Yazici, Ahmet Atasever, Ozge Cetinarslan, Ebru Turan, Ertan Sagbas, Yıldıray Yuzer

**Affiliations:** ^1^ Department of General Surgery, Faculty of Medicine, Hacettepe University, Ankara, Türkiye; ^2^ Department of General Surgery, Faculty of Medicine, Demiroglu Bilim University, İstanbul, Türkiye; ^3^ Department of Cardiology, Faculty of Medicine, Demiroglu Bilim University, İstanbul, Türkiye; ^4^ Faculty of Medicine, Demiroglu Bilim University, İstanbul, Türkiye; ^5^ Department of Cardiovascular Surgery, İstanbul Florence Nightingale Hospital, İstanbul, Türkiye; ^6^ Department of General Surgery, İstanbul Florence Nightingale Hospital, İstanbul, Türkiye

**Keywords:** end stage liver disease (ESLD), coronary artery disease, living donor liver transplant (LDLT), OPCAB (off pump coronary artery bypass), liver, transplantation

## Abstract

**Background:**

End-stage liver disease (ESLD) patients frequently exhibit comorbid coronary artery disease (CAD), complicating liver transplantation (LT) due to increased perioperative cardiovascular risk. In patients for whom percutaneous coronary intervention (PCI) is not feasible, coronary artery bypass grafting (CABG) may be required prior to or during LT. Off-pump CABG (OPCAB) presents a promising strategy to minimize the hemodynamic and inflammatory burdens associated with cardiopulmonary bypass, especially in ESLD patients undergoing major surgery.

**Case presentations:**

We present two male patients (aged 60 and 61) with ESLD and significant LAD stenosis who underwent simultaneous OPCAB and living donor liver transplantation (LDLT). The first case involved cryptogenic cirrhosis and recurrent variceal bleeding; the second had HBV/HDV-related cirrhosis and hepatocellular carcinoma. In both cases, OPCAB was performed using the left internal mammary artery (LIMA) graft on a beating heart. Subsequently, LDLT was carried out using standard piggy-back techniques. Portal pressure modulation via splenic artery ligation was performed in the first case due to elevated post-reperfusion portal flow. Anesthetic management emphasized hemodynamic monitoring and stability. Both patients were extubated on postoperative day one, discharged with triple immunosuppression, and followed for 6–12 months with preserved cardiac and graft function. A bile leak from the cystic duct anastomosis was encountered in one case.

**Conclusion:**

Simultaneous OPCAB and LDLT is a feasible and safe approach in carefully selected ESLD patients with CAD when performed by experienced multidisciplinary teams. Avoiding PCI mitigates bleeding risks associated with dual antiplatelet therapy, while OPCAB circumvents the deleterious effects of cardiopulmonary bypass. This strategy may shorten transplant wait times and optimize both cardiac and hepatic outcomes in high-risk populations.

## Introduction

End-stage liver disease (ESLD) has been associated with increased cardiovascular risk factors (1). The prevalence of coronary artery disease (CAD) in individuals with end-stage liver disease (ESLD) has been reported to range from 2.5% to 27%, with the highest rates seen in patients over 50 years of age (2, 3).

Existing literature demonstrates that coronary artery disease significantly contributes to poor prognosis and elevated mortality rates among patients with liver cirrhosis, particularly those awaiting liver transplantation (4). Advancements in surgical and medical technology have made liver transplantation (LT) a viable option for older patients and those in high-risk categories (5). The high prevalence of coronary artery disease and its adverse effects in patients with end-stage liver disease necessitates a multidisciplinary approach. In particular, planning surgical intervention in this complex patient group requires careful evaluation. In recent years, innovative procedures such as simultaneous off-pump coronary artery bypass surgery (OPCAB) and living donor liver transplantation (LDLT) have been shown to be successfully performed in high-risk patients (6).

In this study, we present two patients who underwent OPCAB and LDLT in the same session for both coronary artery disease and end-stage liver disease, and aim to share our experience regarding the clinical management of this complex procedure.

## Case reports

### Case 1

A 61-year-old male patient with a known history of diabetes mellitus presented to our clinic with cryptogenic liver cirrhosis. The patient was classified as Child-Pugh Class A, and his model for end-stage liver disease (MELD) score was calculated as 10 at the time of admission. Despite undergoing multiple sessions of band ligation, the patient had persistent esophageal variceal bleeding and refractory ascites. Additionally, he had a history of smoking and reported symptoms of exertional dyspnea and exercise intolerance.

**Figure 1:**
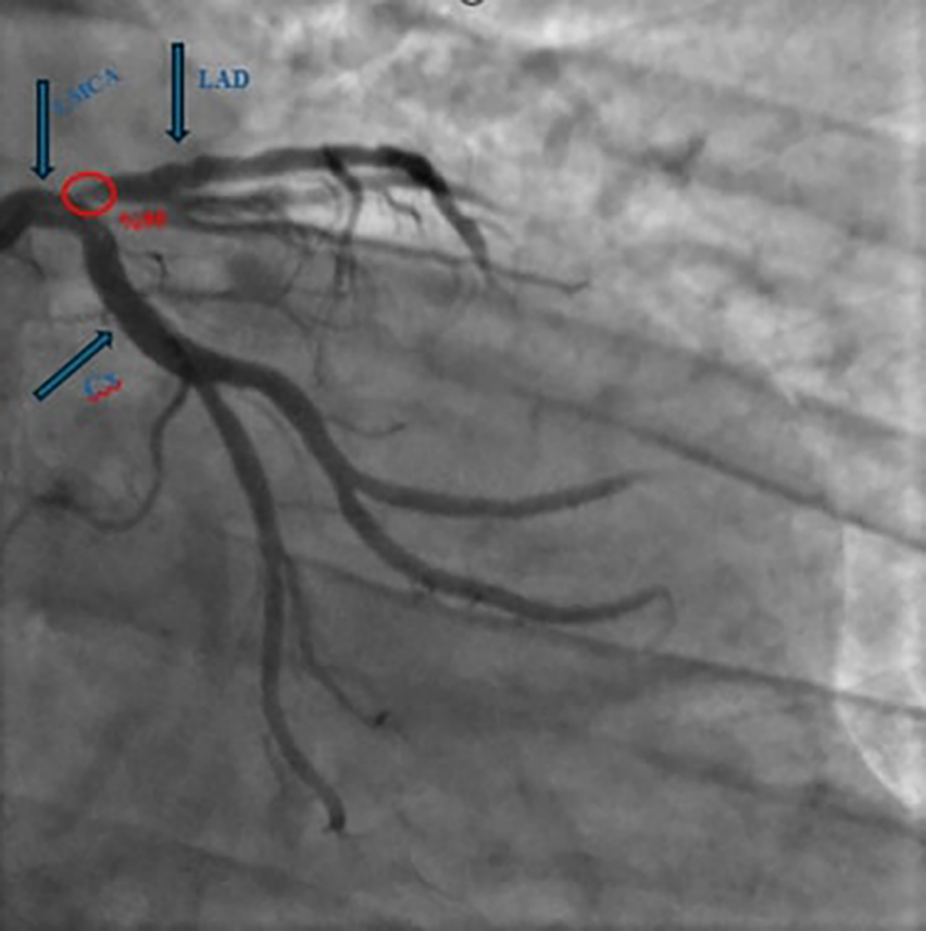
Coronary angiography revealed a 95% stenosis in the left anterior descending artery. (LAD: left anterior descending artery, LMCA: left main coronary artery, Cx: circumflex arteries).

Cardiac evaluation revealed mild mitral regurgitation, an ejection fraction of 53%, and a systolic pulmonary artery pressure of 29 mmHg on transthoracic echocardiography. The presence of apical hypokinesia on echocardiography prompted coronary angiography without the need for further ischemic testing. Coronary angiography revealed a 95% stenosis in the left anterior descending artery (LAD) (Figure 1).

### Operative procedure and technical details for case 1

Under non-invasive monitoring and general anesthesia, the surgical procedure began with off-pump coronary artery bypass grafting (OPCAB). Following a median sternotomy, the left internal mammary artery (LIMA) graft was prepared. After opening the pericardium, a distal anastomosis of the LIMA to the LAD artery was performed on a beating heart using an Octopus stabilizer. During the OPCAB procedure, heparin was administered at half the standard dose to minimize bleeding risks. At the conclusion of the OPCAB procedure, heparin was neutralized with protamine.

The liver transplant phase commenced with donor hepatectomy. Upon confirmation of the suitability of the donor liver, the recipient team initiated recipient hepatectomy through a reverse-T incision. During the procedure, 5 L of ascitic fluid were drained from the peritoneal cavity. After the hilar dissection was completed, perihepatic dissection was performed, mobilizing the caudate and right lobes from the retrohepatic vena cava using the “piggy-back” technique with caval preservation. Following total hepatectomy, an 886-g right liver lobe graft was prepared for implantation. End-to-end hepatic venous anastomosis was performed between the recipient's right hepatic vein stump and the donor's right hepatic vein using a continuous, intraluminal everting technique. Portal venous anastomosis was performed end-to-end between the recipient's main portal vein and the donor's right portal vein using the same technique. The arterial anastomosis was completed under a microscope, and biliary reconstruction was performed using the duct-to-duct technique. Portal pressure was measured as 25 mmHg after reperfusion. To prevent graft over-inflow, the gastrocolic ligament was opened, and the splenic artery was identified and ligated posterior to the pancreas. Following this intervention, the portal pressure decreased to 16 mmHg. Postoperative Doppler ultrasonography revealed a portal flow rate of 1,450 ml/min, confirming successful hemodynamic optimization. At the conclusion of the procedure, hemostasis was confirmed, and the operation was successfully completed.

### Case 2

A 60-year-old male patient with no known prior comorbidities presented to our clinic. The patient had a history of hepatitis B infection for 25 years and hepatitis D virus (HDV) infection for 15 years, resulting in liver cirrhosis. The patient was classified as Child-Pugh Class B, and his model for end-stage liver disease (MELD) score was calculated as 15 at the time of admission. He had no history of ascites, hepatic encephalopathy, or esophageal variceal bleeding. Imaging studies during routine follow-up revealed subcapsular lesions consistent with hepatocellular carcinoma (HCC), measuring 22 mm in diameter in segment 4A and 34 mm in diameter in segment 6 as well as revealing liver cirrhosis.

**Figure 2:**
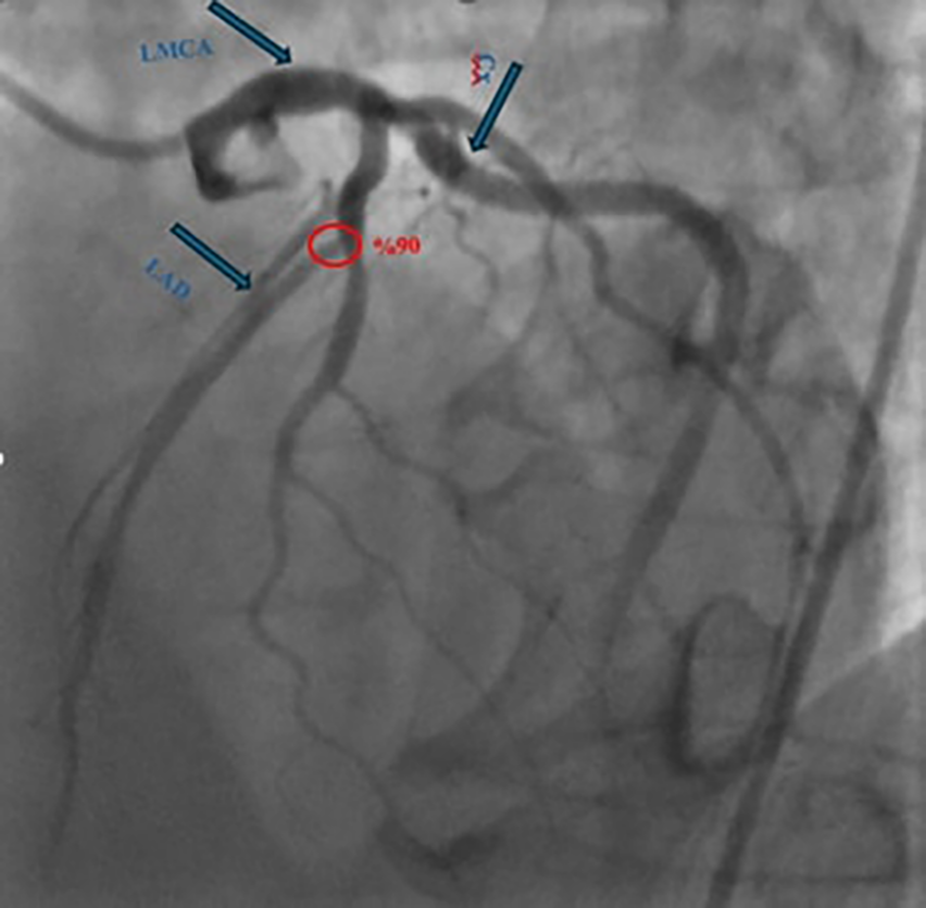
Coronary angiography demonstrated a 90% stenosis at the left anterior descending artery's diagonal branch bifurcation. (LAD: left anterior descending artery, LMCA: left main coronary artery, Cx: circumflex arteries).

The patient reported no active cardiac symptoms. However, during the preoperative evaluation, routine dobutamine stress echocardiography revealed a right bundle branch block, prompting coronary angiography. Coronary angiography demonstrated a 90% stenosis at the LAD's diagonal branch bifurcation (Figure 2).

### Operative procedure and technical details for case 2

**Figure 3:**
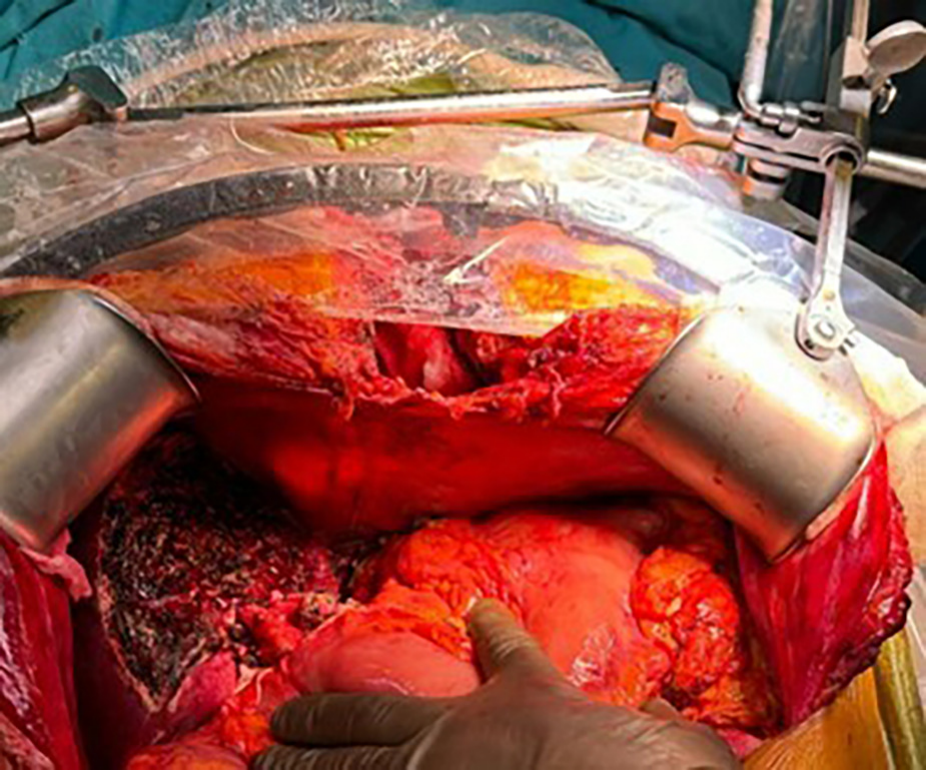
Intraoperatif image of the implanted liver and median sternotomy.

Under non-invasive monitoring and general anesthesia, the surgical procedure began with OPCAB. Following a median sternotomy, the LIMA graft was prepared. After opening the pericardium, a distal anastomosis of the LIMA to the LAD was performed on a beating heart using an Octopus stabilizer. The liver transplant phase commenced with donor hepatectomy. During the recipient hepatectomy phase, 400 ml of ascitic fluid was drained. A 709-g graft was prepared for implantation. The graft had two biliary ducts, necessitating dual biliary reconstructions. One biliary duct was anastomosed to the recipient's common bile duct (duct-to-duct), and the other was anastomosed to the recipient's cystic duct (duct-to-duct). At the conclusion of the procedure, hemostasis was confirmed, and the operation was successfully completed. Intraoperative surgery image is shown in Figure 3. The remaining surgical details are as described in Case 1.

### Anesthesia management

In both patients, anesthesia was carefully tailored to maintain hemodynamic stability during the combined cardiac and transplant procedures. General anesthesia was induced using propofol (1–2 mg/kg), fentanyl (1 mcg/kg), and vecuronium (0.1 mg/kg). Anesthesia maintenance was achieved with a 50% oxygen-air mixture, sevoflurane inhalation, and continuous vecuronium infusion (0.8 µg/kg/min), supplemented with intermittent doses of fentanyl as required. Intraoperative monitoring included central venous pressure (CVP), arterial blood gas analysis, urine output, end-tidal carbon dioxide (EtCO₂), body temperature, and coagulation status assessed via thromboelastography. Advanced hemodynamic monitoring was performed using continuous transesophageal echocardiography (TEE) was utilized throughout both the cardiac and liver transplantation phases to assess myocardial function and guide fluid and inotropic management. This multimodal anesthetic approach aimed to optimize organ perfusion, minimize fluid overload, and prevent perioperative hemodynamic instability, which is critical in patients with ESLD undergoing complex surgical interventions.

### Postoperative follow-up and clinical outcomes

The surgical procedures for both patients were completed uneventfully. The operation duration for the first patient was 6.5 h, whereas the second patient's operation lasted 10 h due to the prolonged biliary anastomosis. During the surgeries, the first patient received a total of 4 units of erythrocyte suspension, while the second patient required 6 units of erythrocyte suspension. Both patients were extubated without complications on postoperative day 1 and oral intake was initiated. Postoperatively, both patients were started on triple immunosuppression therapy consisting of steroids, tacrolimus, and mycophenolate mofetil. Acetylsalicylic acid was also administered to both patients for antithrombotic management. The first patient has been followed for 12 months postoperatively with good liver and cardiac function. The second patient has been under follow-up for 6 months. During this period, the second patient experienced a bile leak from the cystic duct anastomosis, which was managed by the placement of a Percutaneous Transhepatic Biliary Drainage (PTBD). Demographic characteristics of the patients and perioperative data are presented in Table 1. Comprehensive cardiologic evaluations, including transthoracic echocardiography and standard electrocardiography, were conducted at 1 and 6 months post-discharge. Both assessments revealed no evidence of ischemia, arrhythmia, or structural cardiac abnormalities.

**Table 1:** Demographic characteristics of the patients and perioperative data.

Perioperative data	Case 1	Case 2
Age/gender	61 years old/male	60 years old/male
Body mass index	24.4	26.9
CHILD score	A	B
MELD score	10	15
Ethiology of cirrhosis	Cryptogenic	HBV + HDV + HCC
Intraoperative bleedıng time	1,100	900
Intraoperative erythrocyte suspention given	4 units	6 units
Duration of operation	6.5 h	10 h
Postoperative follow-Up	12 months	6 months
Postoperative complications	Uneventful	Bile leakage
Duration of hospital stay	25 days	35 days

## Discussion

CAD poses a significant problem in patients scheduled for LT. CAD has a major impact on surgical outcomes and may increase perioperative mortality and morbidity, especially in ESLD (7). Patients with ischemic heart disease due to CAD often have limited cardiac reserve and even minor hemodynamic disturbances can lead to serious intraoperative complications (8).

In certain patient populations, percutaneous coronary intervention (PCI) may not be a feasible therapeutic approach. Under these circumstances, open-heart surgery becomes a requisite intervention, even in the presence of significant comorbidities. Evidence from a systematic review comparing PCI and coronary artery bypass grafting (CABG) in individuals with multivessel CAD indicates that CABG demonstrates superior efficacy in lowering mortality rates (9).

Studies suggest that 6 months of dual antiplatelet therapy is appropriate in chronic coronary syndrome after PCI, regardless of stent type. Longer continuation of dual antiplatelet therapy is preferable for patients at high thrombotic risk but low bleeding risk, while for patients whom are at high bleeding risk, the duration of therapy may be limited to 1–3 months (10).

In patients undergoing simultaneous liver transplantation (LT) and coronary artery disease (CAD) intervention, choosing between coronary artery bypass grafting (CABG) and percutaneous coronary intervention (PCI) requires careful consideration. PCI, while less invasive and associated with a shorter recovery time, necessitates the continuation of dual antiplatelet therapy to prevent stent thrombosis.

CABG can be performed before liver transplantation, but may make the operation more difficult due to the increased risk of bleeding associated with chronic liver disease. Long-term follow-up after cardiac surgery in patients with ESLD has shown a higher mortality rate (11).

Our multidisciplinary team, including the specialties of cardiology, cardiothoracic surgery, and transplant surgery, after careful evaluation of the patients' general conditions and available clinical data, predicted that the waiting time for LT could pose a serious risk of mortality and morbidity. In this context, a comprehensive decision-making process was carried out to determine the most appropriate surgical approach for the management of both CAD and ESLD in this patient group, especially considering the effects of antiplatelet therapy on potential bleeding complications in the perioperative period. One of our patients had persistent variceal bleeding and our other patient had a malignancy; therefore, waiting an additional period of time for a transplant or increasing the risk of perioperative bleeding were not feasible options. As a result of these thorough evaluations, we determined that the most appropriate treatment strategy for our patients with CAD was simultaneous CABG and LDLT. This approach provides several advantages, such as shortening the waiting time during the liver transplantation process, minimizing the risk of bleeding associated with perioperative antiplatelet therapy management by refraining from initial PCI, and reducing operational risks by completing two major surgical interventions in a single procedure. CABG was preferred over PCI in order to optimize both cardiac revascularization and transplant outcomes safely.

Recent advancements in surgical methodologies have enabled CABG to be conducted using two distinct approaches. The first, OPCAB, is performed on a beating heart without the utilization of cardiopulmonary bypass (CPB). The second, On-Pump CABG (ONCAB), involves the use of CPB and cardioplegic arrest. Evidence from meta-analyses and extensive research indicates that long-term survival rates over a 10-year period are comparable between OPCAB and ONCAB techniques, with no statistically significant differences observed (12).

ONCAB, which requires cardiopulmonary bypass (CPB), is associated with a significant systemic inflammatory response, increased risk of coagulopathy, renal dysfunction, pulmonary complications, all of which are particularly deleterious in patients with ESLD undergoing LT (13, 14).

In contrast, OPCAB avoids the use of CPB by performing grafting on a beating heart, thus minimizing hemodynamic instability, reducing systemic inflammation, and decreasing the risk of bleeding and end-organ dysfunction (6).

Studies have shown that massive transfusion can adversely affect the recovery of liver function after transplantation and reduce the survival rate after surgery (15, 16). The major advantage of OPCAB surgery compared with ONCAB is that it requires less blood product transfusion and has lower hematologic complications (13).

In the light of these findings, one of the most important reasons why we prefer OPCAB during liver transplantation is that liver transplantation is already a procedure that causes severe inflammatory and hemodynamic stress, and the fact that OPCAB does not require the use of CPB prevents the addition of this stress and offers a more controlled process during the operation. In addition, by reducing the need for transfusion, it may prevent situations that may adversely affect the healing of the liver graft after transplantation (14, 17).

Starting the surgery with off-pump coronary bypass in the simultaneous operation suggests that stabilizing the coronary flow in liver transplantation, which will cause high hemodynamic changes, will reduce myocardial ischemia and cope with intraoperative cardiac stress more effectively (14).

CAD presents significant challenges in the pre-transplant evaluation and management of patients with ESLD. Effective management of CAD is essential to enhance perioperative outcomes and improve long-term survival in this high-risk population.

Recently, Vohra et al. reported a case series of eight patients undergoing combined living donor liver transplantation and coronary artery bypass grafting, in which two patients experienced fatal postoperative events (18). This study highlights the potential morbidity associated with the combined approach. Therefore, such complex procedures should be limited to high-volume centers equipped with specific technical and organizational capacities, and with established access to living donor liver transplantation programs.

## Conclusion

A multidisciplinary approach and individualized surgical planning in patients with ESLD and CAD enable the implementation of innovative strategies, such as liver transplantation combined with OPCAB, providing a safe and effective treatment option when paired with meticulous patient selection and the expertise of experienced teams.

## Data Availability

The raw data supporting the conclusions of this article will be made available by the authors, without undue reservation.
